# A Machine Learning Approach for Predicting Caco-2 Cell Permeability in Natural Products from the Biodiversity in Peru

**DOI:** 10.3390/ph17060750

**Published:** 2024-06-07

**Authors:** Victor Acuña-Guzman, María E. Montoya-Alfaro, Luisa P. Negrón-Ballarte, Christian Solis-Calero

**Affiliations:** Faculty of Pharmacy and Biochemistry, Universidad Nacional Mayor de San Marcos, Lima 15001, Peru

**Keywords:** Caco-2 cell line, permeability, intestinal absorption, bioavailability, machine learning, molecular descriptor, drug discovery, natural products

## Abstract

Background: Peru is one of the most biodiverse countries in the world, which is reflected in its wealth of knowledge about medicinal plants. However, there is a lack of information regarding intestinal absorption and the permeability of natural products. The human colon adenocarcinoma cell line (Caco-2) is an in vitro assay used to measure apparent permeability. This study aims to develop a quantitative structure–property relationship (QSPR) model using machine learning algorithms to predict the apparent permeability of the Caco-2 cell in natural products from Peru. Methods: A dataset of 1817 compounds, including experimental log Papp values and molecular descriptors, was utilized. Six QSPR models were constructed: a multiple linear regression (MLR) model, a partial least squares regression (PLS) model, a support vector machine regression (SVM) model, a random forest (RF) model, a gradient boosting machine (GBM) model, and an SVM–RF–GBM model. Results: An evaluation of the testing set revealed that the MLR and PLS models exhibited an RMSE = 0.47 and R^2^ = 0.63. In contrast, the SVM, RF, and GBM models showcased an RMSE = 0.39–0.40 and R^2^ = 0.73–0.74. Notably, the SVM–RF–GBM model demonstrated superior performance, with an RMSE = 0.38 and R^2^ = 0.76. The model predicted log Papp values for 502 natural products falling within the applicability domain, with 68.9% (*n* = 346) showing high permeability, suggesting the potential for intestinal absorption. Additionally, we categorized the natural products into six metabolic pathways and assessed their drug-likeness. Conclusions: Our results provide insights into the potential intestinal absorption of natural products in Peru, thus facilitating drug development and pharmaceutical discovery efforts.

## 1. Introduction

Peru is one of the most biodiverse countries in the world, because it possesses over 70% of the planet’s biodiversity, which is represented in a variety of ecosystems [1]. The richness of this diversity is reflected in the country’s wealth of knowledge about medicinal plants, which has been passed down by several generations for treating various conditions [2]. Some examples include *Uncaria tomentosa* (“Uña de gato”), which is used for treating various health problems, such as gastric ulcers, viral infections, arthritis, and other inflammatory problems [3]; *Plukenetia volubilis* (“Sacha inchi”), which is traditionally used for skin care to soften skin, heal wounds, and treat insect bites and skin infections [4]; and *Hypericum laricifolium* (“Hierba de la fortuna”), which used for inflammation, infections, and musculoskeletal and bone pain [5]. However, there is a lack of information regarding the pharmacological properties, safety, and efficacy of natural products present in natural resources used in oral administration [6,7].

Natural products are organic compounds obtained from natural resources, such as plants, animals, and microorganisms that exhibit biological activities [8,9]. Historically, they have been used as the primary source of compounds for medicines, cosmetics, and food products [10]. They can be classified into primary and secondary metabolites [11]. Secondary metabolites are compounds that may provide an evolutionary advantage due to the organism adapting to its environment [8,11], and they are derived from biosynthetic intermediates, such as acetyl coenzyme A, shikimic acid, mevalonic acid, and 1-deoxyxylulose-5-phosphate [8,9]. Natural products can be categorized into six metabolic pathways: terpenoids, polyketides, fatty acids, alkaloids, shikimates and phenylpropanoids, and amino acids and peptides [12].

The gastrointestinal tract is a channel lined with epithelium that extends from the mouth to the anus and is responsible for food digestion, nutrient absorption, waste product excretion, and water reabsorption [13]. The absorptive region, which includes the stomach, small intestine, and part of the large intestine, is composed of a layer of epithelial cells called enterocytes [13]. Enterocytes represent the largest population of intestinal cells, presenting an apical membrane facing the gastrointestinal lumen and a basolateral membrane facing the serosal side [13,14]. The intestinal barrier separates the intestinal lumen from the internal host, facilitating the exchange of molecules and nutrient absorption, while preventing water and electrolyte loss, and the entry of antigens and microorganisms into the body [15,16].

The efficacy of oral drug administration relies on intestinal absorption of the compound [17]. According to the Biopharmaceutics Classification System, aqueous solubility and permeability are properties that influence oral absorption [18]. Intestinal permeability is a property that describes the facility with which molecules pass through the intestinal epithelium via simple passive diffusion [13]. It is classified into effective permeability or the human jejunal permeability rate, which is determined from intestinal perfusion studies [19], and the apparent permeability (Papp), which is the amount of compound transported per unit of time. 

The human colon adenocarcinoma cell line (Caco-2) is commonly used as an in vitro assay to measure apparent permeability, due to its physiological and morphological properties, similar to enterocytes [20]. However, this assay is time consuming due to the long culture periods between 21 and 24 days, making it challenging to perform high-throughput screening [21]. An alternative approach for predicting apparent permeability in the Caco-2 cell line is quantitative structure–property relationship (QSPR) modeling. It is a mathematical model that correlates experimental values with chemical structure features to predict the properties of new chemical entities [22]. Chemical structure features are represented by numerical physicochemical parameters called molecular descriptors. Molecular descriptors are determined experimentally or computationally, and are utilized in the development QSPR models [22]. 

Machine learning (ML) is a technique that uses algorithms to analyze data, learn from it, and then make predictions about new datasets [23]. ML is widely used in all stages of drug discovery, including ligand identification, target validation, and identification of prognostic biomarkers [23]. ML is divided into supervised learning methods, which are used to develop training models to predict future values, and unsupervised learning methods, which enable the clustering of data [23]. ML models have been utilized in QSPR studies to predict the apparent permeability of Caco-2 cells. One of the pioneering efforts was that by Norinder et al. [24], who developed a PLS model using 17 compounds. Since this initial study, the availability of experimental data has increased, leading to a growth in QSPR studies aimed at predicting Caco-2 cell permeability, involving varying in data sizes, descriptor types and quantities, and modeling methods. Various types of descriptors, encompassing both 2D and 3D molecular descriptors, have been utilized, and a range of regression algorithms have been employed, including multiple linear regression (MLR), partial least squares (PLS), support vector machine (SVM), random forest (RF) [20,21,25,26,27], and gradient boosting [28].

There is a need to obtain more pharmacological information on natural products, such as apparent permeability, which is highly correlated with intestinal absorption and determines the efficacy of orally administered compounds. This study focuses on the development of QSPR models using ML algorithms to correlate experimental values on the apparent permeability in the Caco-2 cell line with calculated molecular descriptors. Then, the best-performing QSPR model is utilized to predict the apparent permeability of natural products from the biodiversity in Peru. These findings will allow the pharmacokinetic and pharmacodynamic properties of natural products to be inferred, such as absorption and potential bioavailability, providing insights into the compounds responsible for the pharmacological activity of natural resources.

## 2. Results and Discussion

### 2.1. Analysis of the Datasets

**Figure 1:**
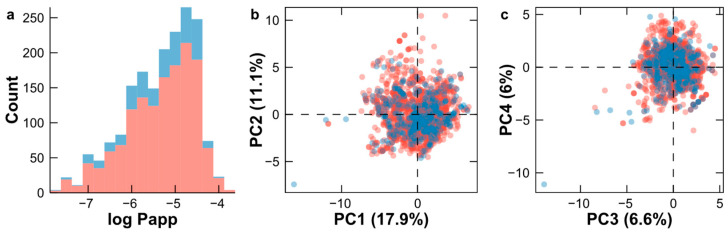
Analysis of modeling dataset: (**a**) distribution of log Papp values for the training and testing sets; (**b**) PCA plot for PC1 and PC2 based on 41 selected descriptors; (**c**) PCA plot for PC3 and PC4 based on 41 selected descriptors. Training set (red) and testing set (blue).

In this study, we employed a dataset source from the literature [28] comprising of 1817 unique compounds. The diversity of the dataset was evaluated based on five physicochemical properties: molecular weight (MW) (437.95 ± 152), logP (1.56 ± 2.0), number of hydrogen acceptors (7.24 ± 3.8), number of hydrogen donors (2.81 ± 2.6), topological surface area (TPSA) (106.51 ± 59.3), and number of rotatable bonds (6.23 ± 4.3). The log Papp values exhibited a mean of −5.34, ranging from a minimum of −7.70 to a maximum of −3.78. Both the training and testing sets exhibited a comparable distribution of log Papp values, as illustrated in Figure 1a. Additionally, principal component analysis (PCA) was performed using 41 selected descriptors. The first 40 principal components (PCs), which explained 99.8% of the cumulative variance, were plotted in pairs along the x-axis and y-axis (Figure S3). Furthermore, the first four PCs are plotted in Figure 1b,c. This analysis validated the suitability of the partitioning of our datasets, as the chemical space for both the training and testing sets exhibited a similarity. Consequently, the testing set, utilized for external validation, adequately represented the distribution of the log Papp values and chemical space present in the training set.

### 2.2. Feature Selection and Development of QSPR Models

To evaluate the impact of our feature selection approach, we developed six QSPR models: MLR, PLS, SVM, RF, GBM, and SVM–RF–GBM. These models were constructed using three different datasets: a dataset without feature selection (523 predictors), a dataset with RFE (60 predictors), and a dataset with both RFE and GA (41 predictors). We evaluated the performance of each model using RMSE and R^2^ metrics for the training set, cross-validation, and testing set to evaluate the performance of each model (Tables S5, S6 and Table 1). Superior model performance is indicated by low RMSE values and high R^2^ values, while inferior performance is characterized by high RMSE values and low R^2^ values. Our results indicated that the performance metrics across the three datasets were quite similar for each model. This suggests that our feature selection approach effectively reduced the model complexity by eliminating less important predictors, while preserving the predictive performance. Consequently, we selected the RFE–GA approach, which identified 41 molecular descriptors for subsequent analysis.

**Figure 2:**
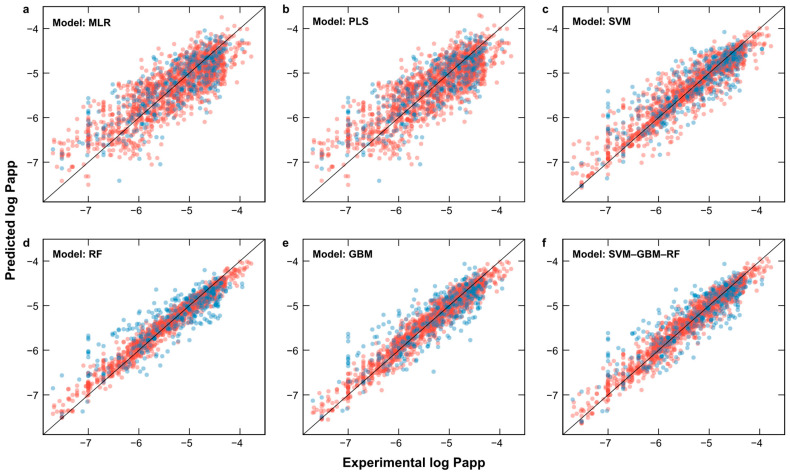
Experimental and predicted log Papp values: (**a**) multiple linear regression (MLR); (**b**) partial least squares (PLS); (**c**) support vector machine (SVM); (**d**) random forest (RF); (**e**) gradient boosting machine (GBM); (**f**) ensemble SVM–GBM–RF model. Red circles represent the training set; blue circles represent the testing set.

The MLR and PLS models exhibited similar predictive capabilities, as observed from their RMSE and R^2^ values across the training, testing, and cross-validation sets. Both models demonstrated the lowest performance, with the highest RMSE and lowest R^2^ values (RMSE_Test_ = 0.47 and R^2^_Test_ = 0.63). The SVM, RF, and GBM models showed enhanced predicted performance (RMSETest = 0.40, 0.39, and 0.39, respectively, R^2^ = 0.73, 0.74, and 0.74, respectively). To further improve the predictive capacity, an ensemble of SVM, RF, and GBM was utilized to create the SVM–RF–GBM model, which exhibited superior performance (RMSE_Test_ = 0.38 and R^2^ = 0.76) and was selected for subsequent analysis (Figure 2 and Table S7). These results suggest that the apparent permeability in Caco-2 cells is influenced by multiple variables with non-linear relationships. The SVM–RF–GBM model demonstrates a predictive capability comparable to that reported by Wang and Chen [28], with similar R^2^ values and a marginally lower RMSE in this study (Table 1), despite employing different modeling approaches. These findings imply that there is a multivariate explanation for apparent permeability, as it is influenced by 41 molecular descriptors.

### 2.3. Mechanism Interpretation

In accordance with the OECD guidelines, a QSPR study should include mechanism interpretation whenever possible. Apparent permeability quantifies the transport rate of a compound across Caco-2 cells over time, facilitating the inference of pharmacological parameters, such as absorption and bioavailability [29].

Table 2 reveals that the top ten descriptors that are predominantly associated with hydrogen bonds, including maxHBint7, maxHBint5, maxHBint9, maxHBint3, Eta_D_epsiD, and maxHBd, consistent with the findings by Wang and Chen [28]. Additionally, descriptors related to lipophilicity, such as AlogP and electrotopological state indices (E-state) [30], offer electronic and topological insights into the molecular structure. E-state indices are classified into four groups: E-state sums, Atom-type counts, the E-state minimum, and the E-state maximum [30,31].

According to the QSPR SVM–RF–GBM model, the top ten molecular descriptors exhibit moderate to low linear correlation (Table 2) with the experimental log Papp values, as indicated by the Pearson correlation coefficient (r). The importance of the variables for each model are detailed in the supplementary information (Figures S4 and S5). Furthermore, the 41 molecular descriptors demonstrated moderate, low, or no linear correlation with the experimental log Papp values, as depicted in the scatter plot (Figures S6 and S7). Additionally, outliers are observed.

### 2.4. Applicability Domain of the SVM–RF–GBM Model

**Figure 3:**
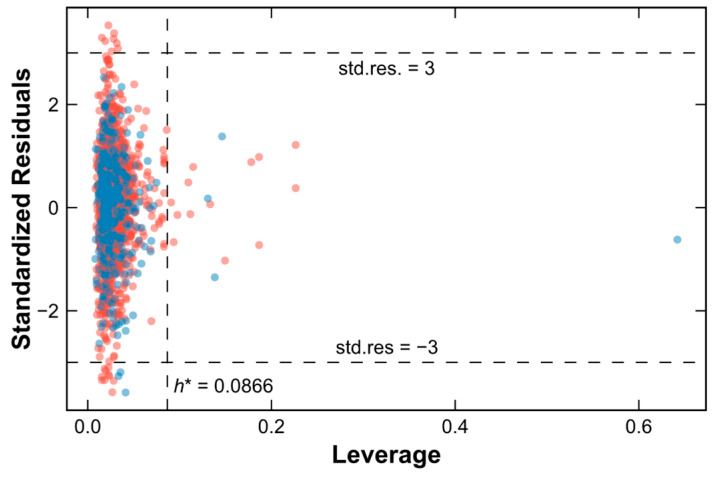
Williams plot. Red circles represent the training set; blue circles represent the testing set. h*: warning leverage.

The applicability domain determines the compounds for which the log Papp predictions may not be reliable, in accordance with the third criterion in the OECD principles [32]. Leverage values for both the training and testing sets were obtained by calculating the hat matrix of the training set. The Williams plot exhibited x-axis leverages and y-axis standardized residuals. The horizontal dashed lines indicate a standard residual of ±3, while the vertical dashed line denotes the warning leverage, h* = 0.0866, indicating the limits of the applicability domain. According to the Williams plot, 38 outliers were identified outside the applicability domain, 31 (2.13%) outliers of which were from the training set and 7 (1.93%) of which were from the testing set (Figure 3).

### 2.5. Comparative Analysis of the SVM–RF–GBM Model and the Other Models

We compared our results with those of previous QSPR investigations targeting the prediction of log Papp values in Caco-2 cells (Table 3). This comparison encompassed details such as the methodology employed, molecular descriptors utilized, number size, and RMSE_Test_ and R^2^_Test_ values. Our methodology used a combination of 2D and 3D descriptors sourced from PaDEL-Descriptor and alvaDesc, drawing from a substantial dataset comprising 1817 compounds. In contrast, the study conducted by Wang and Chen [28] employed a dual-RBF neural network model utilizing only 2D descriptors, achieving a similar performance, with an RMSE_Test_ of 0.39 and an R^2^_Test_ of 0.76. Other investigations featured QSPR models constructed with smaller sample sizes, thereby limiting the diversity of the compounds amenable to prediction.

### 2.6. Prediction of Log Papp Values for Natural Products from Peru

**Figure 4:**
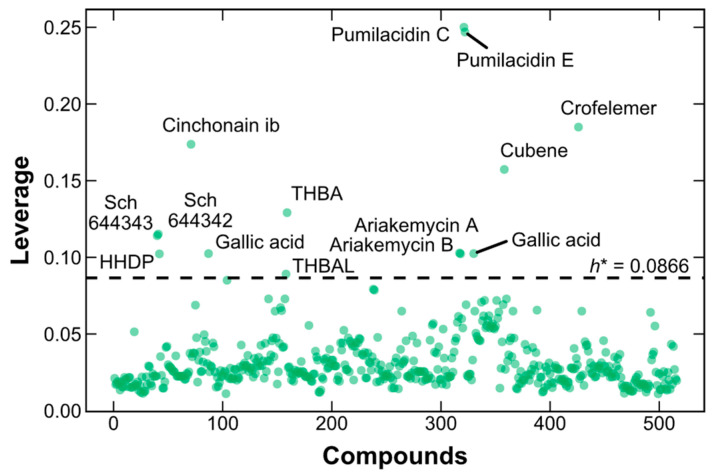
Leverage plot of natural product database from Peru. Horizontal dashed line: warning leverage, h* = 0.0866.

The SVM–RF–GBM model predicted the log Papp values of 516 natural products sourced from the biodiversity in Peru. However, 14 compounds were excluded due to falling outside the applicability domain based on the warning leverage, h* = 0.0866, which defines the boundary. One duplicate compound (gallic acid) was removed as it appeared in two different articles. Subsequently, the remaining 13 compounds outside the applicability domain were removed (Figure 4): SCH 644343, SCH 644342, hexahydroxydiphenic acid (HHDP), cinchonain Ib, gallic acid, 2,4,6-trihydroxybenzaldehyde (THBAL), 2,4,6-trihydroxybenzoic acid (THBA), ariakemycin A, ariakemycin B, pumilacidin C, pumilacidin E, cubene, and crofelemer. The final dataset, used for further analysis, comprised 502 compounds.

**Figure 5:**
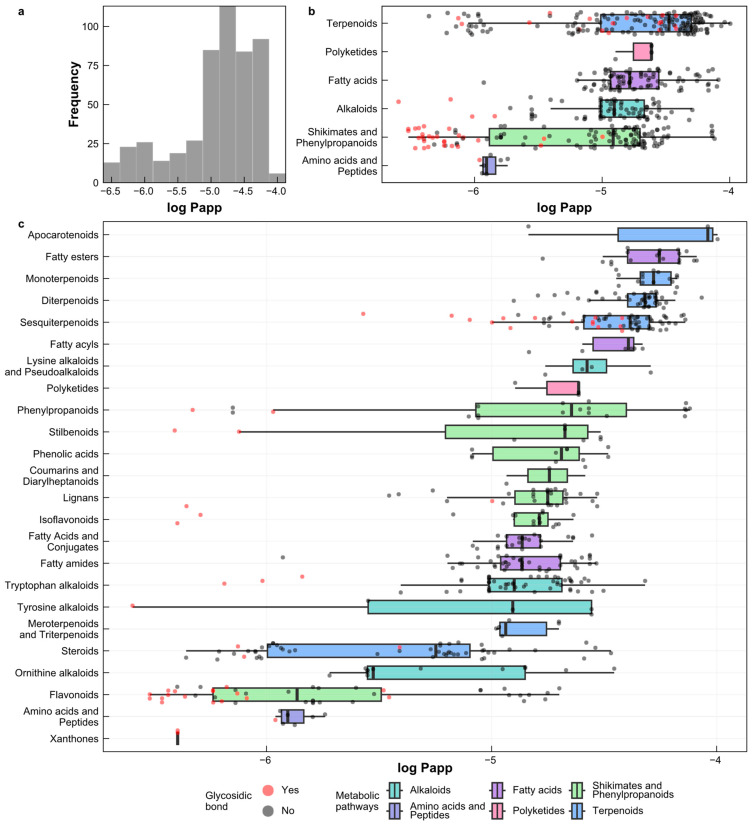
(**a**) Distribution of predicted log Papp values in natural products; (**b**) distribution of predicted log Papp values by chemical groups; (**c**) distribution of predicted log Papp values by metabolic pathway. Red circles represent the presence of a glycosidic bond; black circles indicate the absence of a glycosidic bond.

The distribution of the log Papp values revealed that 68.93% (n = 346) of the compounds exhibited values greater than −5, classifying them as having high intestinal absorption [33]. Another 21.51% (n = 108) fell within the range between −6 to −5, indicating medium intestinal absorption. Finally, 9.56% (n = 48) of the compounds had Log Papp values below −6, suggesting low intestinal absorption. The log Papp value distribution showed a mean log Papp value of −4.91, with values ranging from −6.59 (minimum) to −3.99 (maximum) (Figure 5a).

The natural products were classified into six metabolic pathways: alkaloids, fatty acids, polyketides, terpenoids, shikimates and phenylpropanoids, and amino acids and peptides (Figure 5b). The natural products showed a diverse distribution of the log Papp values in each pathway, encompassing high, medium, and low apparent permeability. For a clearer overview of the compounds characterized by high permeability, each metabolic pathway was further subdivided into chemical groups, with the exception of polyketides and amino acids and peptides. Upon analyzing these chemical groups, 11 groups primarily exhibited high apparent permeability (log Papp > −5) [33] (Figure 5c). These groups included apocarotenoids, fatty esters, monoterpenoids, diterpenoids, sesquiterpenoids, fatty acyls, lysine alkaloids and pseudoalkaloids, and polyketides, indicating the potential for high intestinal absorption via passive transport. Additionally, eight groups showed a distribution of log Papp values primarily in the medium to high permeability range, encompassing phenylpropanoids, stilbenoids, phenolic acids, coumarins and diarylheptanoids, lignans, isoflavonoids, fatty acids and conjugates, and fatty amides. The remaining eight groups demonstrated a mixed distribution, with compounds falling into low, medium, and high log Papp categories, including tryptophan alkaloids, tyrosine alkaloids, meroterpenoids and triterpenoids, steroids, ornithine alkaloids, flavonoids, amino acids and peptides, and xanthones.

In the terpenoid pathway, apocarotenoids, meroterpenoids, diterpenoids, and sesquiterpenoids primarily exhibited high apparent permeability. In the fatty acid pathway, fatty esters and fatty acyls showed high permeability. Phenolic compounds, which constitute the largest group of secondary metabolites in plants, are primarily derived from the shikimate and phenylpropanoid pathway [34]. These compounds exhibited distributions of low, medium, and high apparent permeability, aligning with the chemical diversity of this group. Furthermore, we identified 50 compounds containing glycosidic bonds, with the majority falling into the low permeability category (n = 28), followed by the medium permeability category (n = 9), and the high permeability category (n = 13). 

For a compound to cross the intestinal barrier, it must possess a certain degree of lipophilicity, while maintaining solubility in water. Compounds need to be dissolved in an aqueous medium to pass through the lipid bilayer of enterocytes. Some examples of natural products displaying high apparent permeability include α-ionone, R-(−)-Linalool, and methylcativate. These compounds are characterized by a predominance of hydrophobic regions, such as methyls, cyclohexanes, alkanes, and alkenes, facilitating the crossing of the lipid bilayer. Additionally, they contain few polar groups like carbonyl, hydroxyl, and ester groups, enabling the formation of hydrogen bonds with water molecules and providing solubility in an aqueous medium. Conversely, compounds with low apparent permeability, such as betacyanin, luteolin-7-O-glucoside, and genistin, are marked by the presence of numerous polar groups, such as carboxyl, hydroxyl, ether, and carbonyl, alongside fewer hydrophobic regions, leading to low lipophilicity and, subsequently, reducing the ability to cross the lipid bilayer [35].

### 2.7. Evaluation of Drug-Likeness of Natural Products

Assessing the drug-likeness of natural products is a pivotal step for predicting their potential as orally administered drug. The apparent permeability in Caco-2 cell lines serves as an indicator of intestinal absorption, a critical factor in the efficacy of orally administrated medications. Permeability is characterized by various physicochemical properties, including the molecular weight (MW), the octanol–water partition coefficient (logP), the number of hydrogen bond donors (HBDs), the number of hydrogen bond acceptors (HBAs), the topological surface area (TPSA), and the number of rotatable bonds (RBNs), all of which are known to influence intestinal permeability [36,37]. The Lipinski rule of five (Ro5) [36] is a well-established set of criteria utilized for assessing permeability in oral drugs, encompassing parameters such as MW ≤ 500, ClogP ≤ 5 (or MLogP ≤ 4.15), HBD ≤ 5, and HBA ≤ 10. Veber et al. [37] expanded upon these criteria, including TPSA ≤ 140 and RBN ≤ 10.

**Figure 6:**
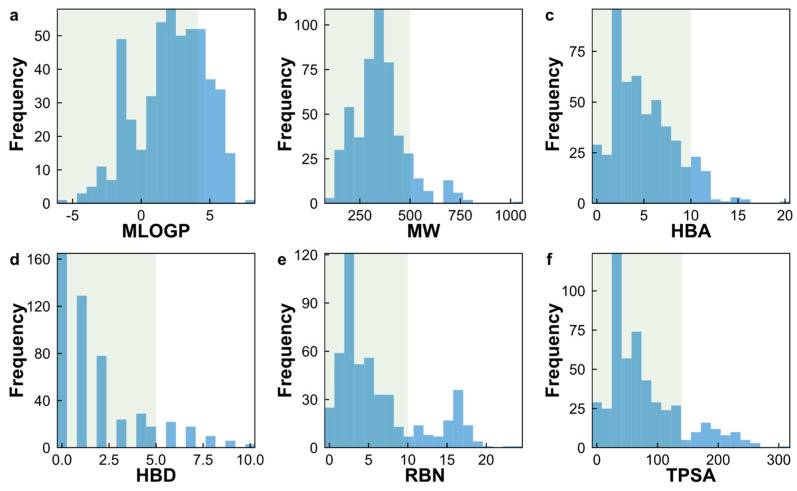
Histogram of six physicochemical properties for a set of 504 natural products sourced the biodiversity in Peru: (**a**) MlogP; (**b**) MW; (**c**) HBA; (**d**) HBD; (**e**) RBN; and (**f**) TPSA. The compliant Lipinski and Veber areas are shown in green.

**Figure 7:**
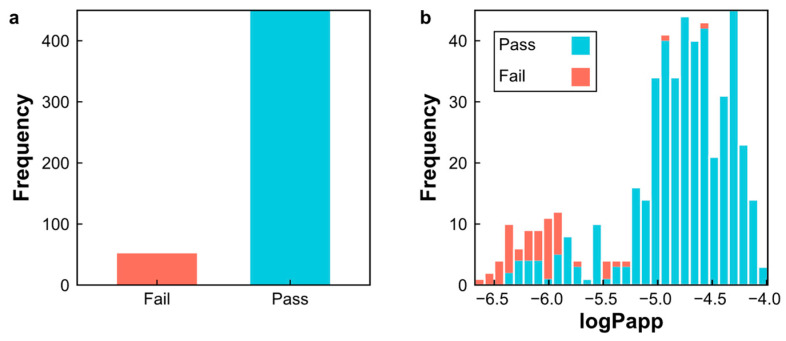
(**a**) Bar chat depicting the compliance of natural products with Ro5; (**b**) distribution of predicted log Papp values among natural products categorized according to their compliance with Ro5.

In this study, we characterized our natural product dataset using these six physicochemical descriptors (Figure 6). About 75.5% (n = 374) of the natural products exhibited an MlogP < 5, 90.0% (n = 452) had an MW ≤ 500, 90.4% (n = 454) met the criteria for a HBA ≤ 10, 88.2% (n = 443) satisfied a HBD ≤ 5, 79.4% (n = 399) possessed an RBN ≤ 10, and 86.4% (n = 434) adhered to a TPSA ≤ 140. Moreover, 90% (n = 450) of the natural products in our dataset conformed to the Ro5 requirement for oral bioavailability, while 10% (n = 52) fell outside the Ro5 boundary in regard to at least one molecular property (Figure 7a). These results suggest that the majority of natural products in our dataset are suitable for oral administration.

Additionally, 99.4% (n = 344) of the natural products with high apparent permeability (log Papp > −5) conformed to the Ro5 requirement (Figure 7b), further supporting their suitability for oral administration and validating the predicted log Papp values generated by our SVM–RF–GBM model for crossing the intestinal barrier.

To gain a deeper understating of compounds well suited for oral administration based on the predicted log Papp values of our natural product dataset, we employed eight drug-likeness scores (*DLS*s). These indices are defined as the ratio between the number of satisfied rules (*nRules*) and the total number of rules (*tRules*) (Equation (1)) [38], as follows:

(1) $$DLS=\frac{nRules}{tRules}$$

- DLS_01 is derived from a modified version of Ro5 [36], comprising four rules, according to Equation (1);
- DLS_02 is defined by six rules, including a HBD ≤ 5, a HBA in the range 1–8, an MW in the range 200–450, an MlogP in the range −2.0–4.5, an RBN in the range from 1–9, and the number of rings ≤ 5 [39];
- DLS_03 incorporates criteria such as a HBD ≤ 5, a HBA ≤ 10, an MW from 200 to 500, an MlogP in the range −5.0–5.0, an RBN ≤ 8, and a formal charge in the range −2–2 [40];
- DLS_04 is defined by parameters including a HBD ≤ 5, a HBA in the range 2–10, an MW in the range 78–500, an MlogP in the range −0.5–5.0, the ratio of the number of C_sp3_ atoms to the total number of non-halogen atoms in the range 0.15–0.8, the ratio of the number of hydrogen atoms to the total number of non-halogen atoms in the range 0.6–1.6, and the ratio of molecular unsaturation (Unsat-p) in the range 0.10–0.45 [41];
- DLS_05 relies on criteria such as the ratio of the total number of oxygen and nitrogen atoms and the number of C_sp3_ atoms in the range 0.10–1.80, and a descriptor Unsat-p ≤ 0.43 [42];
- DLS_06 is based on a HBD ≤ 5, a HBA ≤ 10, an MW ≤ 500, an MlogP ≤ 5, an RBN ≤ 10, and a TPSA ≤ 140 [43];
- DLS_07 is based on the criteria of an RBN ≤ 10, and a TPSA ≤ 140 [37];
- DLS_cons represents the average drug-likeness score obtained from the previously described criteria.

**Figure 8:**
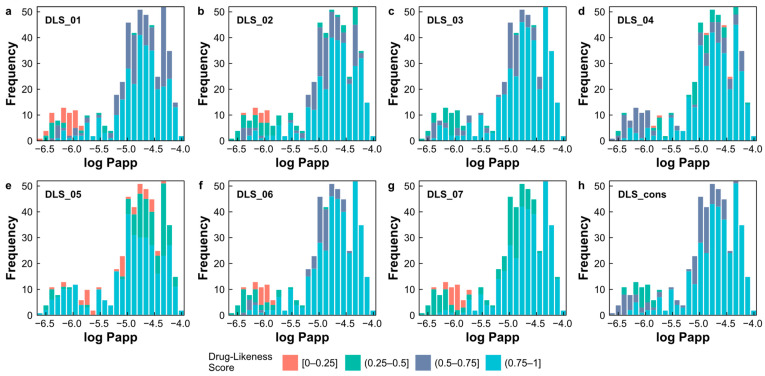
(**a**–**h**) Distribution of predicted log Papp values grouped by eight drug-likeness score indexes. Scores are divided into four categories: [0–0.25], (0.25–0.5], (0.5–0.75], and (0.75–1].

We found that the majority of natural products with high apparent permeability (log Papp > −5) exhibited drug-likeness scores within the range of 0.75 to 1, indicating their potential as drugs (Figure 8a–h). Specifically, DLS_cons revealed that 82.7% (n = 286) of the predicted high apparent permeability of the natural products achieved scores within this range. These different approaches to assessing drug-likeness based on various criteria of molecular properties expressed in scores are consistent with the predicted log Papp values generated by the SVM–RF–GBM model, reaffirming its robust predictive capability.

## 3. Materials and Methods

**Figure 9:**
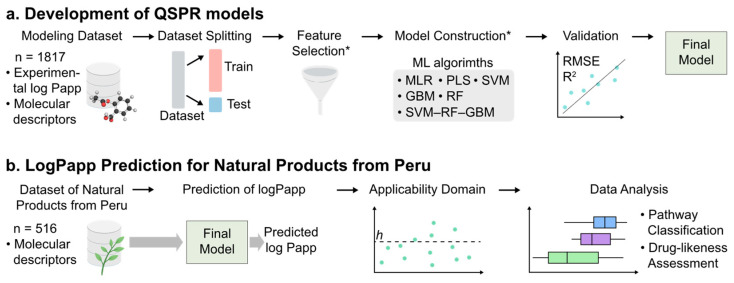
Overview of the workflow of the QSPR models for predicting Caco-2 cell apparent permeability in natural products from Peru. *: The training set was exclusively utilized in these steps.

A workflow illustrating the development of the QSPR models and the predictions involving natural products sourced from the biodiversity in Peru is shown in Figure 9.

### 3.1. Data Collection

A dataset was compiled consisting of molecules with a measured logarithm of apparent permeability (log Papp) in Caco-2 cells and their simplified molecular input line entry specification (SMILES), as reported by Wang and Chen [28]. 

Apparent permeability is measured in vitro in cell lines and is calculated using Equation (2) [19,29].

(2) $$P_{app}=\frac{dQ/dt}{C_{0}\times A}$$

where *Papp* is measured in cm s^−1^; *dQ*/*dt* is the drug permeation rate through the cells or the amount of substance transferred across the membrane per unit of time (µmol s^−1^); *C*_0_ is the initial concentration of the donor compartment (µM); and *A* is the area of the monolayer (cm^2^) [29]. 

Initially, a total of 1827 molecules were obtained. Duplicated molecules were removed based on their SMILES codes, resulting in a refined dataset containing 1817 unique molecules (Table S1).

Natural products sourced from the biodiversity in Peru were gathered from the literature up to 2019, encompassing 48 articles [3,4,5,44,45,46,47,48,49,50,51,52,53,54,55,56,57,58,59,60,61,62,63,64,65,66,67,68,69,70,71,72,73,74,75,76,77,78,79,80,81,82,83,84,85,86,87,88]. The collected data encompassed the SMILES codes, chemical names, organism names, common organism names, and digital object identifiers (DOIs). The pathway classification of natural products was conducted using NPClassifier [12], based on 3 levels, namely the pathway, group, and subgroup, and considered its presence on the glycosidic bond, wherein the SMILES code was used to classified them.

### 3.2. The Calculation and Optimization of the 3D Structure

The generation of 3D coordinates from the SMILES format was performed using the Molconvert software version 24.1.2. Subsequently, hydrogens were added under the condition of pH = 7.4. The 3D structures were then optimized using the steepest descent method with the Merck molecular force field 94 (MMFF 94) [89]. This optimization process was executed using 5000 steps, with the OpenBabel software 3.1.1 [90]. In cases where certain molecules could not be optimized using MMFF 94, the Generalized Amber Force Field (GAFF) [91] method was employed.

### 3.3. Molecular Descriptor Calculation

A total of 7110 molecular descriptors were calculated, encompassing both 2D and 3D molecular descriptors, using the SMILES codes of the compounds. The calculation was carried out using PaDEL-Descriptor 2.21 [92], which contributed 1785 descriptors, and alvaDesc v2.0.16 [38], which provided 5235 descriptors. 

### 3.4. Splitting of the Dataset

In order to prevent overfitting, the dataset was split into a training set and a testing set, maintaining an 80:20 ratio. This partitioning resulted in 1455 molecules in the training set and 362 molecules in the testing set. The training set was employed for model development, while the testing set was reserved for external validation.

### 3.5. Preprocessing

The dataset initially contained missing values, which was addressed by imputing them using the median value. Subsequently, to eliminate uninformative variables and mitigate redundancy, the descriptors with low variance and those displaying high correlations (indicated by a Pearson correlation coefficient absolute value > 0.70) were removed. The remaining dataset contained 521 descriptors. Finally, the descriptors were centered and scaled.

### 3.6. Feature Selection

Feature selection is a set of methods for reducing dimensionality by selecting a subset of variables from the original feature set while eliminating irrelevant, redundant, or noisy features. Consequently, it can lead to enhanced learning performance, reduced computational expenses, and improved model interpretability. 

To achieve this, recursive feature elimination (RFE) was employed to select the most informative molecular descriptors. This method involved an iterative process, where subsets of descriptors ranging from 1 to 200 were evaluated. At each iteration, a random forest model was trained on the selected subset of descriptors, and the performance was assessed using 5-fold cross-validation (CV), which was repeated 20 times to ensure its robustness. RFE involves fitting a random forest model to all the predictors, initially, and then ranking each predictor based on its importance to the model. Subsequently, at each iteration of the feature selection, the least important predictor is removed, and the model is retrained. This iterative process of removing predictors, one by one, continues until the optimal subset of descriptors is determined based on the root mean square error (RMSE) performance metric. This iterative process led to the selection of 60 molecular descriptors (Table S2 and Figure S1). Subsequently, a genetic algorithm (GA) was integrated with a random forest algorithm to further refine the selection. The GA employed a systematic approach inspired by principles of natural selection for modeling. The GA parameters were configured as follows: maximum generations = 100, population per generation = 50, crossover probability = 0.8, mutation probability = 0.1, and elitism = 0. Moreover, 5-fold cross-validation was employed for the model evaluation. The GA process began by initializing a population of potential solutions, where each solution represented a subset of molecular descriptors. Then, the fitness of each solution was evaluated using a fitness function. In this case, the fitness function aimed to minimize the RMSE metric. Through selection, crossover, and mutation operations, the population iteratively evolved over 100 generations (Figure S2). The process terminated after reaching the maximum generation limit, culminating in the identification of a subset of 41 molecular descriptors optimized for modeling. Additionally, principal component analysis (PCA) was conducted to visualize the distribution of the 41 selected descriptors in chemical space.

### 3.7. Modeling 

In this study, six QSPR models were constructed using the training set, incorporating the 41 selected molecular descriptors as independent variables and the log Papp value as the dependent variable (training set and testing set are given in Tables S3 and S4). The models employed in this study are described below.

Multiple linear regression (MLR), a linear model that leverages multiple independent variables to predict one dependent variable, was used [22]. It assumes a linear relationship between the independent variables and the dependent variable. Partial least squares (PLS), a model that transforms original independent variables into principal components or latent variables represented as linear combinations of independent variables, was used [22]. This method is particularly useful when the predictors are highly collinear or when there are more predictors than observations. Support vector machine (SVM), an algorithm suitable for regression and classification problems, was used. During the regression, the input data is transformed into a feature space through a kernel function, finding a hyperplane that best fits the feature space [93]. For this study, a radial basis function kernel was selected. Decision trees are non-parametric algorithms that partition data into smaller regions using a set of splitting rules. These models produce simple rules that are easy to interpret; however, they typically lack predictive performance capabilities compared to more complex algorithms [93]. Random forest (RF) regression, which combines a set of decision trees with good predictive ability and averages their results to enhance the model’s predictive performance, was used [93,94]. Gradient boosting machine (GBM), an algorithm that combines shallow decision trees to facilitate learning and improve prediction accuracy, was also used [93]. Unlike RF models, GBM builds trees sequentially, with each tree correcting the error of its predecessor. Ensemble methods, which integrate the best-performing models to create a new model or meta-model, achieving a predictive performance surpassing that of individual models, were used [93,94]. In this study, a linear model was trained using predicted values from the SVM, RF, and GBM models using the ensemble method, denoted as SVM–RF–GBM. 

During model training, the hyperparameters were adjusted to control the model’s complexity. To assess the impact of the model tuning parameters on the performance and determine the optimal hyperparameter settings, we utilized 5-fold cross-validation, which was repeated five times. The hyperparameters for each model were as follows: MLR, no hyperparameters; PLS, the number of components (ncomp); SVM, sigma (sigma) and cost (C); RF, the number of randomly selected predictors (mtry); GBM, the number of boosting iterations (n.trees) and the max tree depth (interaction.depth); and SVM–RF–GBM, no hyperparameters. The following parameters were employed for model building: MLR, no additional parameters were employed; PLS, ncomp = 40; SVM, epsilon = 0.1, C = 2, and sigma = 0.015; RF, mtry = 18 and number of trees = 500; GBM, n.trees = 100, interaction.depth = 16, shrinkage = 0.1, and the minimum number of observations in the terminal nodes of the trees (n.minobsinnode) = 10.

### 3.8. Model Validation

For model validation, we employed 5-fold cross-validation repeated five times for internal validation, while the testing set was used for external validation. The performance of each model was evaluated using six statistical parameters: the root mean square error of the training set (RMSE_Train_), the coefficient of determination of the training set (R^2^_Train_), the root mean square error of the cross-validation (RMSE_CV_), the coefficient of determination of the cross-validation (R^2^_CV_), the root mean square error of the testing set (RMSE_Test_), and the coefficient of determination of the testing set (R^2^_Test_).

### 3.9. Applicability Domain

The applicability domain of the QSPR model ensures that the models can reasonably and accurately predict uncertain compounds [95]. The leverage values or hat values measure the distance of a data point from the center of the distribution of the training set and are employed to assess whether a compound falls within the domain. The hat values are calculated from the diagonal values in the hat matrix (*H*), which are defined by Equation (3).

(3) $$H=X^{\prime }{\left(X^{\prime }X\right)}^{-1}X$$

In a training set matrix with molecular descriptors of n rows and p columns, $X_{n\times p}$, the hat matrix (*H*) is calculated using Equation (3), where $X^{\prime }$ is the transpose matrix and ${\left(X^{\prime }X\right)}^{-1}$ is the inverse matrix of $\left(X^{\prime }X\right)$.

The applicability domain can be determined by analyzing the leverage and standardize residuals to create a Williams plot. Leverages greater than the warning leverage (h*) indicate that the predicted values are unreliable [95,96]. The warning leverage (h*) is computed as 3 (p + 1)/n, where n is the number of compounds and p is the number of predictors. Furthermore, standardized residuals were computed for both the training and testing sets [96]. However, this approach does not allow for calculating the leverage values for new datasets. Therefore, for a new dataset denoted as a matrix (u), the hat matrix is calculated using Equation (4).

(4) $$h=u^{\prime }{\left(X^{\prime }X\right)}^{-1}u$$

where *X* is the matrix of the training set, u is the matrix of the unknown dataset, and $u^{\prime }$ is the transpose matrix. Finally, the leverage values of the new sample, h, are obtained from the diagonal of the matrix.

In accordance with the OECD guidelines [32], a QSPR study should encompass an applicability domain. The leverage values for the training and testing sets were calculated using the training set of the hat matrix, and standardized residuals were computed for both sets. Subsequently, a Williams plot was constructed using the leverages and standardized residuals [96].

### 3.10. Computational Processing

The computational experiments were conducted at the Center for High Computational Performance of the Peruvian Amazon, affiliated with the Research Institute of the Peruvian Amazon (IIAP) [97]. To perform various essential tasks, the following software tools were employed: OpenBabel version 3.1.1 was utilized for the addition of hydrogens, structure minimization, and conformer search; Molconvert was used to create 3D structures from the SMILES format; Marvin version 21.20.0; and the R programming language version 4.1.1 [98] was used for data analysis, modeling, validation, and visualization.

## 4. Conclusions

In this study, we established six QSPR models employing ML algorithms to predict Caco-2 cell apparent permeability: MLR, PLS, SVM, RF, GBM, and SVM–RF–GBM. Utilizing the data of 1817 molecules, a subset of the original database of 1827 molecules, we evaluated the performance of these models. Among them, the SVM–RF–GBM model demonstrated superior performance based on both internal and external validation metrics. This model was further employed to predict the log Papp values for a collection of natural products sourced from the biodiversity in Peru. 

We assembled a comprehensive database comprising 516 natural products from the biodiverse in Peru. These compounds were classified into six metabolic pathways, encompassing alkaloids, fatty acids, polyketides, terpenoids, shikimates and phenylpropanoids, and amino acids and peptides. Furthermore, the SVM–RF–GBM model was utilized to predict the apparent permeability in Caco-2 cells for 516 natural products, where 502 natural products fell within the applicability domain of the model. We found 346 natural products (68.9%) exhibited high permeability, with 344 (99.4%) conforming to the Ro5 criteria, suggesting their potential suitability for oral administration and favorable intestinal absorption. These findings provide valuable insights into the apparent permeability of a diverse array of natural products from the biodiversity in Peru. The development of QSPR models and the compilation of this natural product database offer valuable tools for further research in drug development and pharmaceutical discovery.

## Figures and Tables

**Table 1:** Metrics for training set, testing set, and cross-validation, along with hyperparameters, employing RFE and GA for feature selection.

Model	RMSE_Train_	R^2^_Train_	RMSE_CV_	R^2^_CV_	RMSE_Test_	R^2^_Test_	Hyperparameters
MLR	0.43	0.70	0.44	0.68	0.47	0.63	-
PLS	0.43	0.70	0.44	0.68	0.47	0.63	ncomp = 11
SVM	0.28	0.87	0.40	0.74	0.40	0.73	sigma = 0.015, C = 2
RF	0.16	0.97	0.40	0.75	0.39	0.74	mtry = 18
GBM	0.19	0.94	0.40	0.74	0.39	0.74	n.trees = 100, interaction.depth = 16
SVM–RF–GBM	0.19	0.94	0.38	0.76	0.38	0.76	-

**Table 2:** The ten best molecular descriptors according to the SVM–RF–GBM model.

Molecular Descriptor	Group	r	Description
maxHBint7	E-state	−0.50	Maximum E-state descriptors of strength for potential hydrogen bonds of path length 7
ALogP	Constitucional	0.46	Ghose-Crippen LogKow
SpMAD_Dzs	Barysz matrix	−0.43	Spectral mean absolute deviation from Barysz matrix/weighted by I-state
maxHBint5	E-state	−0.48	Maximum E-state descriptors of strength for potential hydrogen bonds of path length 5
maxHBint9	E-state	−0.47	Maximum E-state descriptors of strength for potential hydrogen Bonds of path length 9
Eta_D_epsiD	ETA index	−0.39	Eta measure of hydrogen bond donor atoms
maxHBint3	E-state	−0.46	Maximum E-state descriptors of strength for potential hydrogen bonds of path length 3
SHED_DL	Pharmacophore descriptor	−0.47	SHED donor–lipophilic
maxHBd	E-state	−0.35	Maximum E-states for (strong) hydrogen bond donors
Hypertens.80	Drug-like index	0.41	Ghose–Viswanadhan–Wendoloski antihypertensive-like index at 80%

r: Pearson’s correlation coefficient.

**Table 3:** Comparative results of QSPR studies.

Study	Method	Descriptors	N	RMSE_Test_	R^2^_Test_	Reference
This study	SVM–RF–GBM	2D and 3D descriptors from PaDEL-Descriptor and alvaDesc	1817	0.38	0.76	-
Wang and Chen, 2020	Dual-RBF neural network	2D descriptors from PaDEL-Descriptor	1827	0.39	0.76	[28]
Wang et al., 2016	Boosting	2D and 3D MOE descriptors	1017	0.31	0.812	[21]
Lanevskij et al., 2019	Non-linear least squares	log Do/w, pKa, NHD, Vx	442	0.49	0.77	[20]

## Data Availability

The data presented in this study are available on request from the corresponding author.

## Supplementary Materials

The following supporting information can be downloaded at: https://www.mdpi.com/article/10.3390/ph17060750/s1, Figure S1: Recursive feature elimination for molecular descriptor selection; Figure S2: Genetic algorithm for molecular descriptor selection; Figure S3: Plot of the first 40 principal components (PCs) based on 41 selected descriptors. Figure S4: Importance of top ten molecular descriptors in QSPR models; Figure S5: Relative variable importance chart for the SVM–RF–GBM model; Figures S6 and S7: Correlation between experimental log Papp values and selected molecular descriptors; Table S1: Dataset for modeling; Table S2: Results of recursive feature elimination; Table S3: Training set; Table S4: Testing set; Table S5: Metrics for training set, testing set, and cross-validation, without feature selection; Table S6: Metrics for training set, testing set, and cross-validation, employing RFE for feature selection; and Table S7: Experimental and predicted log Papp values.

## References

[1] MINAM (2014). Estrategia Nacional de Diversidad Biológica al 2021 (Plan de Acción 2014–2018).

[2] Acosta S., Meléndez C. (2013). Catálogo Florístico de Plantas Medicinales Peruanas.

[3] García Giménez D., García Prado E., Sáenz Rodríguez T., Fernández Arche A., De la Puerta R. (2010). Cytotoxic Effect of the Pentacyclic Oxindole Alkaloid Mitraphylline Isolated from *Uncaria* Tomentosa Bark on Human Ewing’s Sarcoma and Breast Cancer Cell Lines. Planta Med..

[4] Wang S., Zhu F., Kakuda Y. (2018). Sacha Inchi (*Plukenetia volubilis* L.): Nutritional Composition, Biological Activity, and Uses. Food Chem..

[5] Guillen Quispe Y., Hwang S., Wang Z., Zuo G., Lim S. (2017). Screening In Vitro Targets Related to Diabetes in Herbal Extracts from Peru: Identification of Active Compounds in *Hypericum laricifolium* Juss. by Offline High-Performance Liquid Chromatography. Int. J. Mol. Sci..

[6] Ahmed I., Leach D.N., Wohlmuth H., De Voss J.J., Blanchfield J.T. (2020). Caco-2 Cell Permeability of Flavonoids and Saponins from *Gynostemma pentaphyllum*: The Immortal Herb. ACS Omega.

[7] Firenzuoli F., Gori L. (2007). Herbal Medicine Today: Clinical and Research Issues. Evid.-Based Complement. Altern. Med..

[8] Bernardini S., Tiezzi A., Laghezza Masci V., Ovidi E. (2018). Natural Products for Human Health: An Historical Overview of the Drug Discovery Approaches. Nat. Prod. Res..

[9] Dias D.A., Urban S., Roessner U. (2012). A Historical Overview of Natural Products in Drug Discovery. Metabolites.

[10] Pilon A.C., Valli M., Dametto A.C., Pinto M.E.F., Freire R.T., Castro-Gamboa I., Andricopulo A.D., Bolzani V.S. (2017). NuBBEDB: An Updated Database to Uncover Chemical and Biological Information from Brazilian Biodiversity. Sci. Rep..

[11] Li J., Larregieu C.A., Benet L.Z. (2016). Classification of Natural Products as Sources of Drugs According to the Biopharmaceutics Drug Disposition Classification System (BDDCS). Chin. J. Nat. Med..

[12] Kim H.W., Wang M., Leber C.A., Nothias L.-F., Reher R., Kang K.B., van der Hooft J.J.J., Dorrestein P.C., Gerwick W.H., Cottrell G.W. (2021). NPClassifier: A Deep Neural Network-Based Structural Classification Tool for Natural Products. J. Nat. Prod..

[13] Ménard S., Cerf-Bensussan N., Heyman M. (2010). Multiple Facets of Intestinal Permeability and Epithelial Handling of Dietary Antigens. Mucosal Immunol..

[14] Xu Y., Shrestha N., Préat V., Beloqui A. (2021). An Overview of in Vitro, Ex Vivo and in Vivo Models for Studying the Transport of Drugs across Intestinal Barriers. Adv. Drug Deliv. Rev..

[15] Bischoff S.C., Barbara G., Buurman W., Ockhuizen T., Schulzke J.-D., Serino M., Tilg H., Watson A., Wells J.M. (2014). Intestinal Permeability—A New Target for Disease Prevention and Therapy. BMC Gastroenterol..

[16] Dahlgren D., Lennernäs H. (2019). Intestinal Permeability and Drug Absorption: Predictive Experimental, Computational and In Vivo Approaches. Pharmaceutics.

[17] Cabrera-Pérez M.Á., Pham-The H. (2018). Computational Modeling of Human Oral Bioavailability: What Will Be Next?. Expert. Opin. Drug Discov..

[18] Amidon G.L., Lennernäs H., Shah V.P., Crison J.R. (1995). A Theoretical Basis for a Biopharmaceutic Drug Classification: The Correlation of in Vitro Drug Product Dissolution and in Vivo Bioavailability. Pharm. Res..

[19] Larregieu C.A., Benet L.Z. (2014). Distinguishing between the Permeability Relationships with Absorption and Metabolism to Improve BCS and BDDCS Predictions in Early Drug Discovery. Mol. Pharm..

[20] Lanevskij K., Didziapetris R. (2019). Physicochemical QSAR Analysis of Passive Permeability Across Caco-2 Monolayers. J. Pharm. Sci..

[21] Wang N.-N., Dong J., Deng Y.-H., Zhu M.-F., Wen M., Yao Z.-J., Lu A.-P., Wang J.-B., Cao D.-S. (2016). ADME Properties Evaluation in Drug Discovery: Prediction of Caco-2 Cell Permeability Using a Combination of NSGA-II and Boosting. J. Chem. Inf. Model..

[22] Dastmalchi S., Hamzeh-Mivehroud M., Sokouti B. (2018). Quantitative Structure—Activity Relationship: A Practical Approach.

[23] Vamathevan J., Clark D., Czodrowski P., Dunham I., Ferran E., Lee G., Li B., Madabhushi A., Shah P., Spitzer M. (2019). Applications of Machine Learning in Drug Discovery and Development. Nat. Rev. Drug Discov..

[24] Norinder U., Österberg T., Artursson P. (1997). Theoretical Calculation and Prediction of Caco-2 Cell Permeability Using MolSurf Parametrization and PLS Statistics. Pharm. Res..

[25] Fredlund L., Winiwarter S., Hilgendorf C. (2017). In Vitro Intrinsic Permeability: A Transporter-Independent Measure of Caco-2 Cell Permeability in Drug Design and Development. Mol. Pharm..

[26] Over B., Matsson P., Tyrchan C., Artursson P., Doak B.C., Foley M.A., Hilgendorf C., Johnston S.E., Lee M.D., Lewis R.J. (2016). Structural and Conformational Determinants of Macrocycle Cell Permeability. Nat. Chem. Biol..

[27] Sherer E.C., Verras A., Madeira M., Hagmann W.K., Sheridan R.P., Roberts D., Bleasby K., Cornell W.D. (2012). QSAR Prediction of Passive Permeability in the LLC-PK1 Cell Line: Trends in Molecular Properties and Cross-Prediction of Caco-2 Permeabilities. Mol. Inf..

[28] Wang Y., Chen X. (2020). QSPR Model for Caco-2 Cell Permeability Prediction Using a Combination of HQPSO and Dual-RBF Neural Network. RSC Adv..

[29] Hubatsch I., Ragnarsson E.G.E., Artursson P. (2007). Determination of Drug Permeability and Prediction of Drug Absorption in Caco-2 Monolayers. Nat. Protoc..

[30] Hall L.H., Mohney B., Kier L.B. (1991). The Electrotopological State: An Atom Index for QSAR. Quant. Struct.-Act. Relat..

[31] Hall L.H., Kier L.B. (1995). Electrotopological State Indices for Atom Types: A Novel Combination of Electronic, Topological, and Valence State Information. J. Chem. Inf. Comput. Sci..

[32] Organisation for Economic Co-operation and Development (2014). OECD Guidance Document on the Validation of (Quantitative) Structure-Activity Relationship [(Q)SAR] Models.

[33] Yee S. (1997). In Vitro Permeability Across Caco-2 Cells (Colonic) Can Predict In Vivo (Small Intestinal) Absorption in Man—Fact or Myth. Pharm. Res..

[34] Marchica A., Cotrozzi L., Detti R., Lorenzini G., Pellegrini E., Petersen M., Nali C. (2020). The Biosynthesis of Phenolic Compounds Is an Integrated Defence Mechanism to Prevent Ozone Injury in *Salvia officinalis*. Antioxidants.

[35] Foye W.O., Lemke T.L., Williams D.A. (2013). Foye’s Principles of Medicinal Chemistry.

[36] Lipinski C.A., Lombardo F., Dominy B.W., Feeney P.J. (2001). Experimental and Computational Approaches to Estimate Solubility and Permeability in Drug Discovery and Development Setting. Adv. Drug Deliv. Rev..

[37] Veber D.F., Johnson S.R., Cheng H.-Y., Smith B.R., Ward K.W., Kopple K.D. (2002). Molecular Properties That Influence the Oral Bioavailability of Drug Candidates. J. Med. Chem..

[38] Mauri A., Roy K. (2020). alvaDesc: A Tool to Calculate and Analyze Molecular Descriptors and Fingerprints. Ecotoxicological QSARs.

[39] Oprea T.I., Gottfries J., Sherbukhin V., Svensson P., Kühler T.C. (2000). Chemical Information Management in Drug Discovery: Optimizing the Computational and Combinatorial Chemistry Interfaces. J. Mol. Graph. Model..

[40] Walters W.P., Murcko M.A. (2002). Prediction of ‘Drug-Likeness’. Adv. Drug Deliv. Rev..

[41] Chen G., Zheng S., Luo X., Shen J., Zhu W., Liu H., Gui C., Zhang J., Zheng M., Puah C.M. (2005). Focused Combinatorial Library Design Based on Structural Diversity, Druglikeness and Binding Affinity Score. J. Comb. Chem..

[42] Zheng S., Luo X., Chen G., Zhu W., Shen J., Chen K., Jiang H. (2005). A New Rapid and Effective Chemistry Space Filter in Recognizing a Druglike Database. J. Chem. Inf. Model..

[43] Rishton G.M. (2003). Nonleadlikeness and Leadlikeness in Biochemical Screening. Drug Discov. Today.

[44] Okuyama E., Umeyama K., Ohmori S., Yamazaki M., Satake M. (1994). Pharmacologically Active Components from a Peruvian Medicinal Plant Huira-Huira (*Culcitium canescens* H. & B.). Chem. Pharm. Bull..

[45] Fuchino H., Koide T., Takahashi M., Sekita S., Satake M. (2001). New Sesquiterpene Lactones from *Elephantopus mollis* and Their Leishmanicidal Activities. Planta Med..

[46] Kang T.-H., Matsumoto K., Tohda M., Murakami Y., Takayama H., Kitajima M., Aimi N., Watanabe H. (2002). Pteropodine and Isopteropodine Positively Modulate the Function of Rat Muscarinic M1 and 5-HT2 Receptors Expressed in *Xenopus oocyte*. Eur. J. Pharmacol..

[47] Tincusi B.M., Jiménez I.A., Bazzocchi I.L., Moujir L.M., Mamani Z.A., Barroso J.P., Ravelo A.G., Hernández B.V. (2002). Antimicrobial Terpenoids from the Oleoresin of the Peruvian Medicinal Plant *Copaifera paupera*. Planta Med..

[48] Hegde V.R., Pu H., Patel M., Das P.R., Butkiewicz N., Arreaza G., Gullo V.P., Chan T.-M. (2003). Two Antiviral Compounds from the Plant *Stylogne cauliflora* as Inhibitors of HCV NS3 Protease. ChemInform.

[49] Hegde V.R., Pu H., Patel M., Black T., Soriano A., Zhao W., Gullo V.P., Chan T.-M. (2004). Two New Bacterial DNA Primase Inhibitors from the Plant *Polygonum cuspidatum*. Bioorg. Med. Chem. Lett..

[50] Hegde V.R., Pu H., Patel M., Das P.R., Strizki J., Gullo V.P., Chou C.-C., Buevich A.V., Chan T.-M. (2004). Three New Compounds from the Plant *Lippia alva* as Inhibitors of Chemokine Receptor 5 (CCR5). Bioorg. Med. Chem. Lett..

[51] Heitzman M.E., Neto C.C., Winiarz E., Vaisberg A.J., Hammond G.B. (2005). Ethnobotany, Phytochemistry and Pharmacology of *Uncaria* (Rubiaceae). Phytochemistry.

[52] Aguayo L., Guzman L., Perez C., Aguayo L., Silva M., Becerra J., Fuentealba J. (2006). Historical and Current Perspectives of Neuroactive Compounds Derived from Latin America. MRMC.

[53] Rojas R., Bustamante B., Ventosilla P., Fernádez I., Caviedes L., Gilman R.H., Lock O., Hammond G.B. (2006). Larvicidal, Antimycobacterial and Antifungal Compounds from the Bark of the Peruvian Plant *Swartzia polyphylla* DC. Chem. Pharm. Bull..

[54] Aguiar C.L., Baptista A.S., Alencar S.M., Haddad R., Eberlin M.N. (2007). Analysis of Isoflavonoids from Leguminous Plant Extracts by RPHPLC/DAD and Electrospray Ionization Mass Spectrometry. Int. J. Food Sci. Nutr..

[55] Castillo D., Arevalo J., Herrera F., Ruiz C., Rojas R., Rengifo E., Vaisberg A., Lock O., Lemesre J.-L., Gornitzka H. (2007). Spirolactone Iridoids Might Be Responsible for the Antileishmanial Activity of a Peruvian Traditional Remedy Made with *Himatanthus sucuuba* (Apocynaceae). J. Ethnopharmacol..

[56] Mesa-Siverio D., Machín R.P., Estévez-Braun A., Ravelo Á.G., Lock O. (2008). Structure and Estrogenic Activity of New Lignans from *Iryanthera lancifolia*. Bioorg. Med. Chem..

[57] Gonzales G.F., Gonzales-Castañeda C. (2009). The Methyltetrahydro-β-Carbolines in Maca (*Lepidium meyenii*). Evid.-Based Complement. Altern. Med..

[58] Kawano M., Otsuka M., Umeyama K., Yamazaki M., Shiota T., Satake M., Okuyama E. (2009). Anti-Inflammatory and Analgesic Components from “Hierba Santa,” a Traditional Medicine in Peru. J. Nat. Med..

[59] Aponte J., Yang H., Vaisberg A., Castillo D., Málaga E., Verástegui M., Casson L., Stivers N., Bates P., Rojas R. (2010). Cytotoxic and Anti-Infective Sesquiterpenes Present in *Plagiochila disticha* (Plagiochilaceae) and *Ambrosia peruviana* (Asteraceae). Planta Med..

[60] Aponte J., Jin Z., Vaisberg A., Castillo D., Málaga E., Lewis W., Sauvain M., Gilman R., Hammond G. (2011). Cytotoxic and Anti-Infective Phenolic Compounds Isolated from *Mikania decora* and *Cremastosperma microcarpum*. Planta Med..

[61] Fuchino H., Kiuchi F., Yamanaka A., Obu A., Wada H., Mori-Yasumoto K., Kawahara N., Flores D., Palacios O., Sekita S. (2013). New Leishmanicidal Stilbenes from a Peruvian Folk Medicine, *Lonchocarpus nicou*. Chem. Pharm. Bull..

[62] Leuner O., Havlik J., Budesinsky M., Vrkoslav V., Chu J., Bradshaw T.D., Hummelova J., Miksatkova P., Lapcik O., Valterova I. (2013). Cytotoxic Constituents of *Pachyrhizus Tuberosus* from Peruvian Amazon. Nat. Prod. Commun..

[63] Wu H., Kelley C.J., Pino-Figueroa A., Vu H.D., Maher T.J. (2013). Macamides and Their Synthetic Analogs: Evaluation of in Vitro FAAH Inhibition. Bioorg. Med. Chem..

[64] Baldera-Aguayo P.A. (2014). Phytochemical Study of *Echinopsis peruviana*. Rev. Soc. Quím Perú..

[65] Hajdu Z., Nicolussi S., Rau M., Lorántfy L., Forgo P., Hohmann J., Csupor D., Gertsch J. (2014). Identification of Endocannabinoid System-Modulating N -Alkylamides from Heliopsis Helianthoides Var. Scabra and *Lepidium meyenii*. J. Nat. Prod..

[66] Reina M., Ruiz-Mesia L., Ruiz-Mesia W., Sosa-Amay F.E., Arevalo-Encinas L., González-Coloma A., Martínez-Díaz R. (2014). Antiparasitic Indole Alkaloids from *Aspidosperma desmanthum* and *A. spruceanum* from the Peruvian Amazonia. Nat. Prod. Commun..

[67] Abderrahim F., Huanatico E., Segura R., Arribas S., Gonzalez M.C., Condezo-Hoyos L. (2015). Physical Features, Phenolic Compounds, Betalains and Total Antioxidant Capacity of Coloured Quinoa Seeds (*Chenopodium quinoa* Willd.) from Peruvian Altiplano. Food Chem..

[68] Colegate S.M., Boppré M., Monzón J., Betz J.M. (2015). Pro-Toxic Dehydropyrrolizidine Alkaloids in the Traditional Andean Herbal Medicine “Asmachilca”. J. Ethnopharmacol..

[69] Esparza E., Hadzich A., Kofer W., Mithöfer A., Cosio E.G. (2015). Bioactive Maca (*Lepidium meyenii*) Alkamides Are a Result of Traditional Andean Postharvest Drying Practices. Phytochemistry.

[70] Girardi C., Fabre N., Paloque L., Ramadani A.P., Benoit-Vical F., González-Aspajo G., Haddad M., Rengifo E., Jullian V. (2015). Evaluation of Antiplasmodial and Antileishmanial Activities of Herbal Medicine *Pseudelephantopus spiralis* (Less.) Cronquist and Isolated Hirsutinolide-Type Sesquiterpenoids. J. Ethnopharmacol..

[71] Patel K., Ruiz C., Calderon R., Marcelo M., Rojas R. (2016). Characterisation of Volatile Profiles in 50 Native Peruvian Chili Pepper Using Solid Phase Microextraction–Gas Chromatography Mass Spectrometry (SPME–GCMS). Food Res. Int..

[72] Schmeda-Hirschmann G., Quispe C., Arana G.V., Theoduloz C., Urra F.A., Cárdenas C. (2016). Antiproliferative Activity and Chemical Composition of the Venom from the Amazonian Toad *Rhinella marina* (Anura: Bufonidae). Toxicon.

[73] Boniface P.K., Baptista Ferreira S., Roland Kaiser C. (2017). Current State of Knowledge on the Traditional Uses, Phytochemistry, and Pharmacology of the Genus *Hymenaea*. J. Ethnopharmacol..

[74] Feuereisen M.M., Zimmermann B.F., Schulze-Kaysers N., Schieber A. (2017). Differentiation of Brazilian Peppertree (*Schinus terebinthifolius* Raddi) and Peruvian Peppertree (*Schinus molle* L.) Fruits by UHPLC–UV–MS Analysis of Their Anthocyanin and Biflavonoid Profiles. J. Agric. Food Chem..

[75] Gálvez Ranilla L., Christopher A., Sarkar D., Shetty K., Chirinos R., Campos D. (2017). Phenolic Composition and Evaluation of the Antimicrobial Activity of Free and Bound Phenolic Fractions from a Peruvian Purple Corn (*Zea mays* L.) Accession. J. Food Sci..

[76] Linares-Otoya L., Linares-Otoya V., Armas-Mantilla L., Blanco-Olano C., Crüsemann M., Ganoza-Yupanqui M., Campos-Florian J., König G., Schäberle T. (2017). Diversity and Antimicrobial Potential of Predatory Bacteria from the Peruvian Coastline. Mar. Drugs.

[77] Quispe Y., Hwang S., Wang Z., Lim S. (2017). Screening of Peruvian Medicinal Plants for Tyrosinase Inhibitory Properties: Identification of Tyrosinase Inhibitors in *Hypericum laricifolium* Juss. Molecules.

[78] Xu Y.-M., Wijeratne E.M.K., Babyak A.L., Marks H.R., Brooks A.D., Tewary P., Xuan L.-J., Wang W.-Q., Sayers T.J., Gunatilaka A.A.L. (2017). Withanolides from Aeroponically Grown *Physalis peruviana* and Their Selective Cytotoxicity to Prostate Cancer and Renal Carcinoma Cells. J. Nat. Prod..

[79] Morales-Soriano E., Kebede B., Ugás R., Grauwet T., Van Loey A., Hendrickx M. (2018). Flavor Characterization of Native Peruvian Chili Peppers through Integrated Aroma Fingerprinting and Pungency Profiling. Food Res. Int..

[80] Stivers N., Islam A., Reyes-Reyes E., Casson L., Aponte J., Vaisberg A., Hammond G., Bates P. (2018). Plagiochiline A Inhibits Cytokinetic Abscission and Induces Cell Death. Molecules.

[81] Alves N.S.F., Setzer W.N., da Silva J.K.R. (2019). The Chemistry and Biological Activities of *Peperomia pellucida* (Piperaceae): A Critical Review. J. Ethnopharmacol..

[82] Carlos Castro J., Dylan Maddox J., Cobos M., Diana Paredes J., Jhoao Fasabi A., Vargas-Arana G., Luis Marapara J., Marcelino Adrianzen P., Zadith Casuso M., Levi Estela S., Perveen S., Al-Taweel A. (2019). Medicinal Plants of the Peruvian Amazon: Bioactive Phytochemicals, Mechanisms of Action, and Biosynthetic Pathways. Pharmacognosy—Medicinal Plants.

[83] Han Y., Chi J., Zhang M., Zhang R., Fan S., Huang F., Xue K., Liu L. (2019). Characterization of Saponins and Phenolic Compounds: Antioxidant Activity and Inhibitory Effects on α-Glucosidase in Different Varieties of Colored Quinoa (*Chenopodium quinoa* Willd). Biosci. Biotechnol. Biochem..

[84] Hwang S.H., Kim H.-Y., Guillen Quispe Y.N., Wang Z., Zuo G., Lim S.S. (2019). Aldose Reductase, Protein Glycation Inhibitory and Antioxidant of Peruvian Medicinal Plants: The Case of *Tanacetum parthenium* L. and Its Constituents. Molecules.

[85] Radice M., Tasambay A., Pérez A., Diéguez-Santana K., Sacchetti G., Buso P., Buzzi R., Vertuani S., Manfredini S., Baldisserotto A. (2019). Ethnopharmacology, Phytochemistry and Pharmacology of the Genus *Hedyosmum* (Chlorantaceae): A Review. J. Ethnopharmacol..

[86] Tauchen J., Huml L., Bortl L., Doskocil I., Jarosova V., Marsik P., Frankova A., Clavo Peralta Z.M., Chuspe Zans M.-E., Havlik J. (2019). Screening of Medicinal Plants Traditionally Used in Peruvian Amazon for in Vitro Antioxidant and Anticancer Potential. Nat. Prod. Res..

[87] Zhang Y., Zhang S., Fan W., Duan M., Han Y., Li H. (2019). Identification of Volatile Compounds and Odour Activity Values in Quinoa Porridge by Gas Chromatography–Mass Spectrometry. J. Sci. Food Agric..

[88] Zhong J.-L., Yan H., Xu H.-D., Muhammad N., Yan W.-D. (2019). Preparation from *Lepidium Meyenii* Walpers Using High-Speed Countercurrent Chromatography and Thermal Stability of Macamides in Air at Various Temperatures. J. Pharm. Biomed. Anal..

[89] Halgren T.A., Nachbar R.B. (1996). Merck Molecular Force Field. IV. Conformational Energies and Geometries for MMFF94. J. Comput. Chem..

[90] O’Boyle N.M., Banck M., James C.A., Morley C., Vandermeersch T., Hutchison G.R. (2011). Open Babel: An Open Chemical Toolbox. J. Cheminform..

[91] Wang J., Wolf R.M., Caldwell J.W., Kollman P.A., Case D.A. (2004). Development and Testing of a General Amber Force Field. J. Comput. Chem..

[92] Yap C.W. (2011). PaDEL-Descriptor: An Open Source Software to Calculate Molecular Descriptors and Fingerprints. J. Comput. Chem..

[93] Boehmke B., Greenwell B.M. (2019). Hands-on Machine Learning with R.

[94] Müller A.C., Guido S. (2016). Introduction to Machine Learning with Python: A Guide for Data Scientists.

[95] Zhu L., Zhao J., Zhang Y., Zhou W., Yin L., Wang Y., Fan Y., Chen Y., Liu H. (2018). ADME Properties Evaluation in Drug Discovery: In Silico Prediction of Blood–Brain Partitioning. Mol. Divers..

[96] Shi Y. (2021). Support Vector Regression-Based QSAR Models for Prediction of Antioxidant Activity of Phenolic Compounds. Sci. Rep..

[97] Instituto de Investigaciones de la Amazonía Peruana Centro de Alto Rendimiento Computacional de la Amazonia Peruana 2017. https://www.iiap.gob.pe/web/manati.aspx/.

[98] R Core Team (2022). R: A Language and Environment for Statistical Computing.

