# Supplementary material for: A Machine Learning Approach for Predicting Caco-2 Cell Permeability in Natural Products from the Biodiversity in Peru
Source: Pharmaceuticals (Basel). 2024 Jun 7;17(6):750. doi: 10.3390/ph17060750 (PMC11206960; doi:10.3390/ph17060750)
Supplement: Supplementary file 1 [file pharmaceuticals-17-00750-s001.zip › Figure S1-S7.pdf]

A machine learning approach for predicting Caco-2 cell permeability in natural products from the biodiversity of Peru

Victor Acuña-Guzman [1], Maria E. Montoya-Alfaro [1], Luisa P. Negron-Ballarte [1] and Cristian Solis-Calero [1,*]

[1] Faculty of Pharmacy and Biochemistry, Universidad Nacional Mayor de San Marcos, Lima 15001, Peru
* Correspondence: csolisc@unmsm.edu.pe

Supplementary Materials

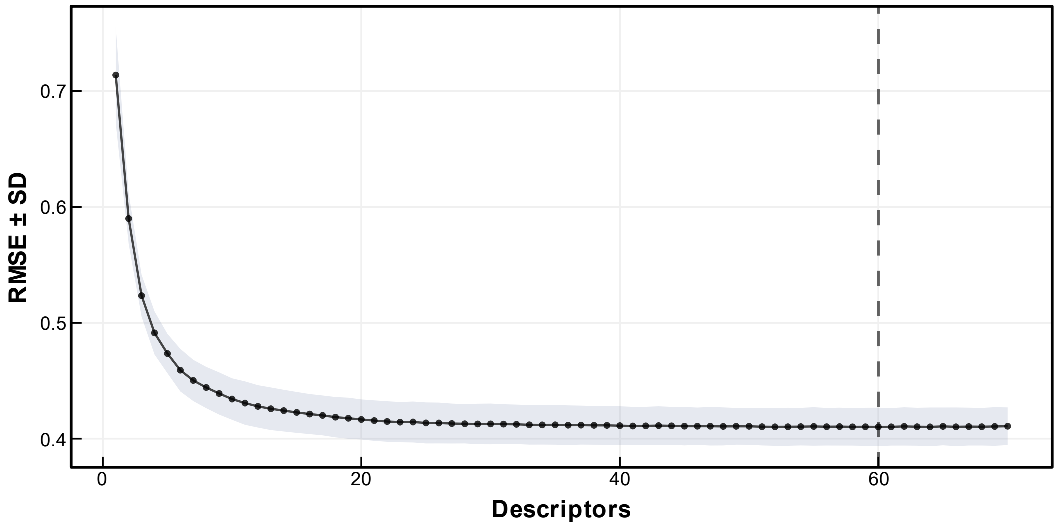

Figure S1. Recursive feature elimination analysis

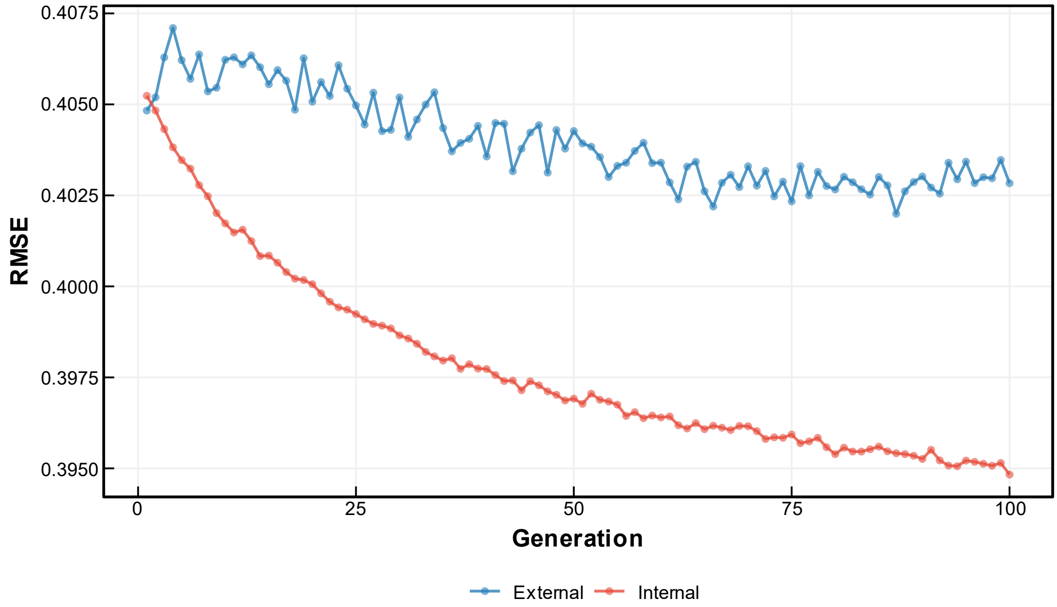

Figure S2. Genetic algorithm optimization

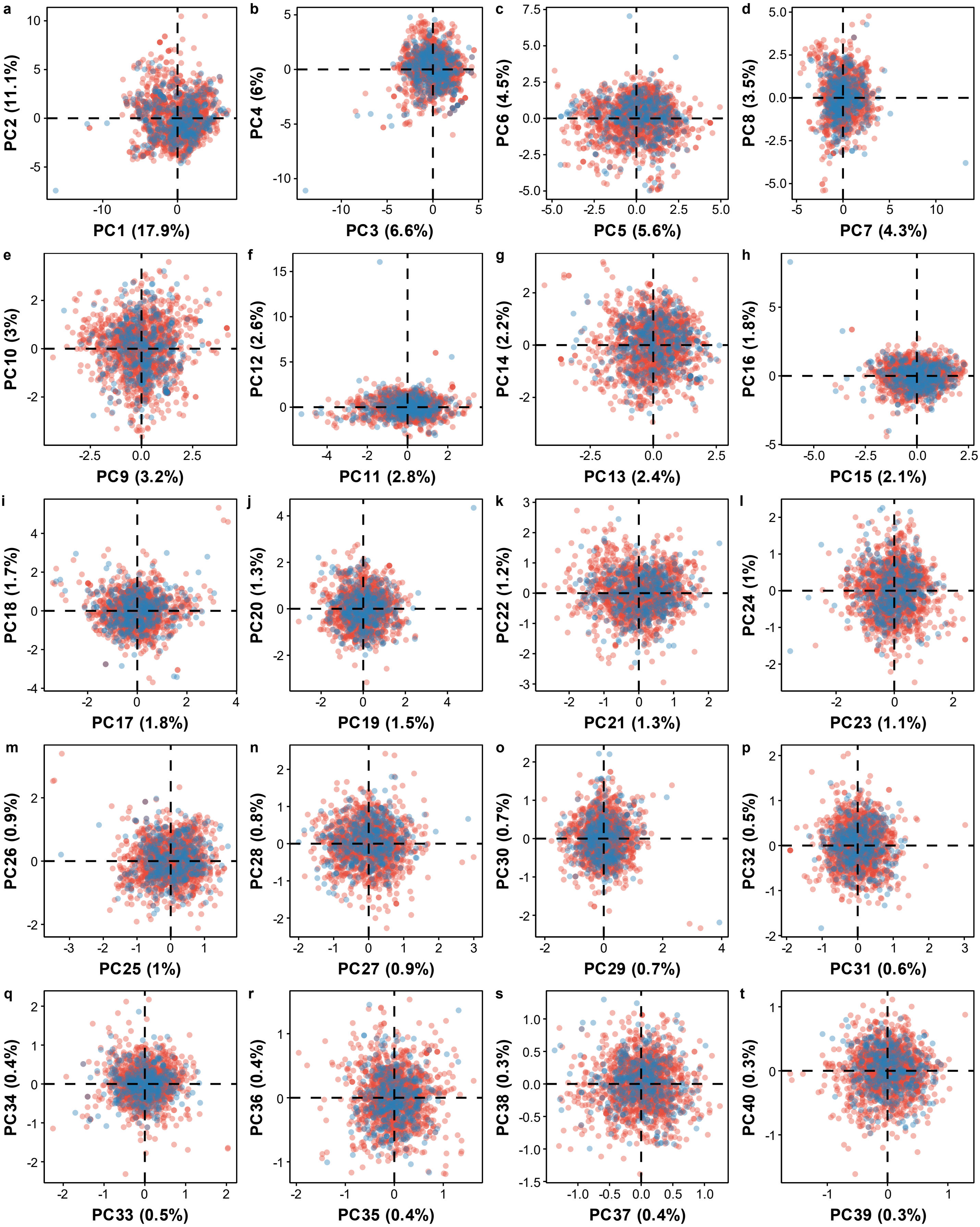

Figure S3. Plot of the first 40 Principal Components (PCs) based on 41 selected descriptors. Training set (red) and testing set (blue)

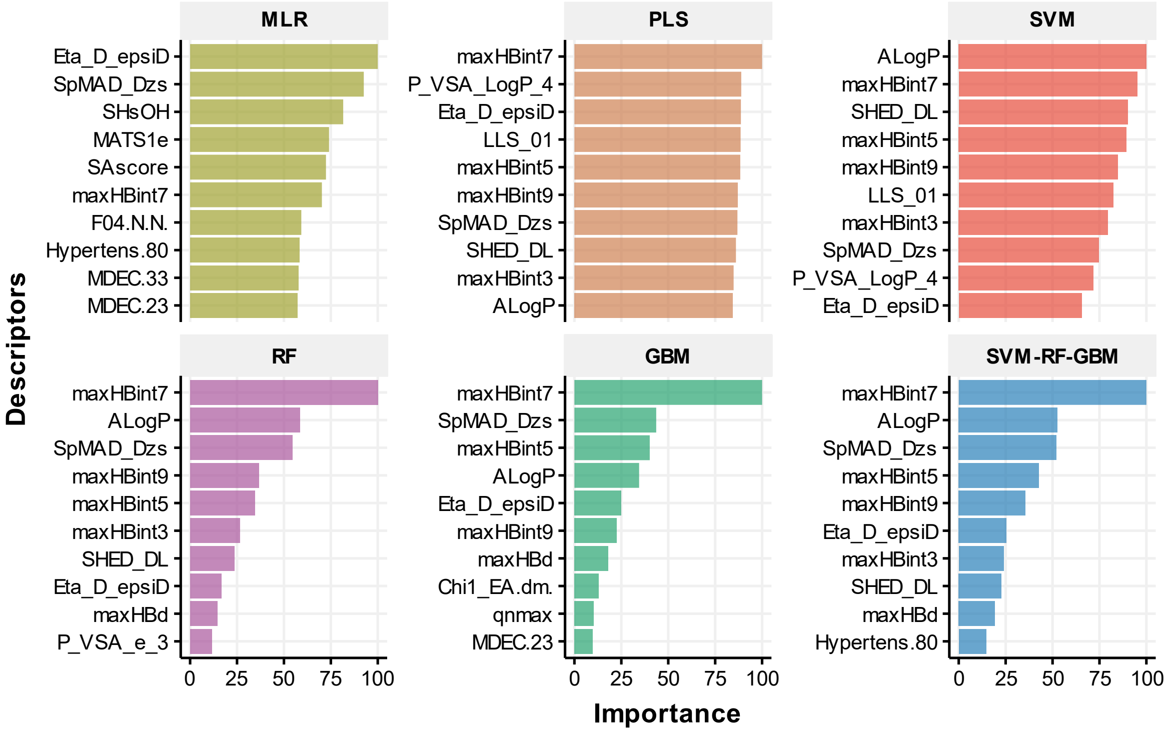

Figure S4. Importance ranking of top ten molecular descriptors for each QSPR model

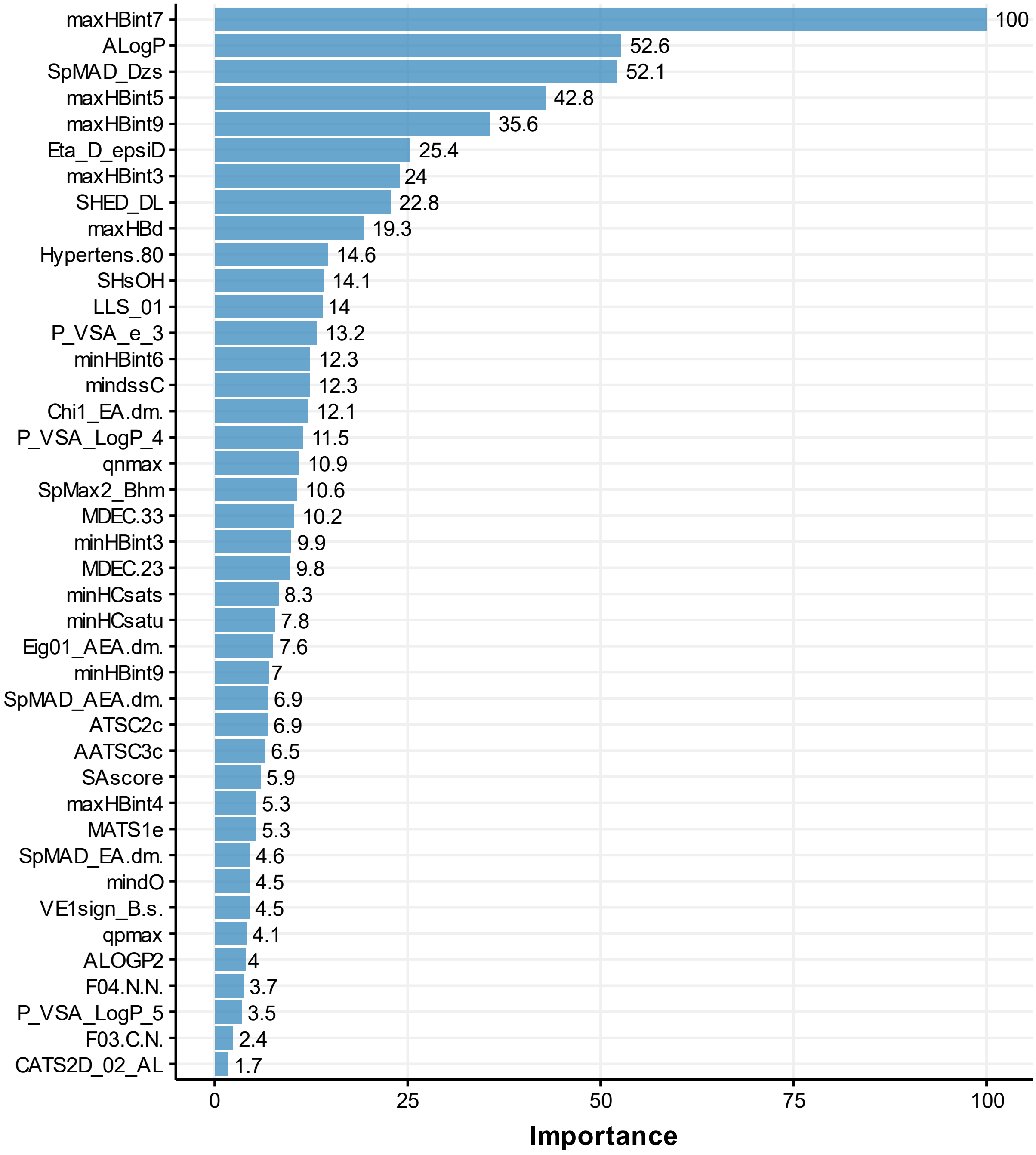

Figure S5. Relative variable importance analysis of the SVM-RF-GBM model

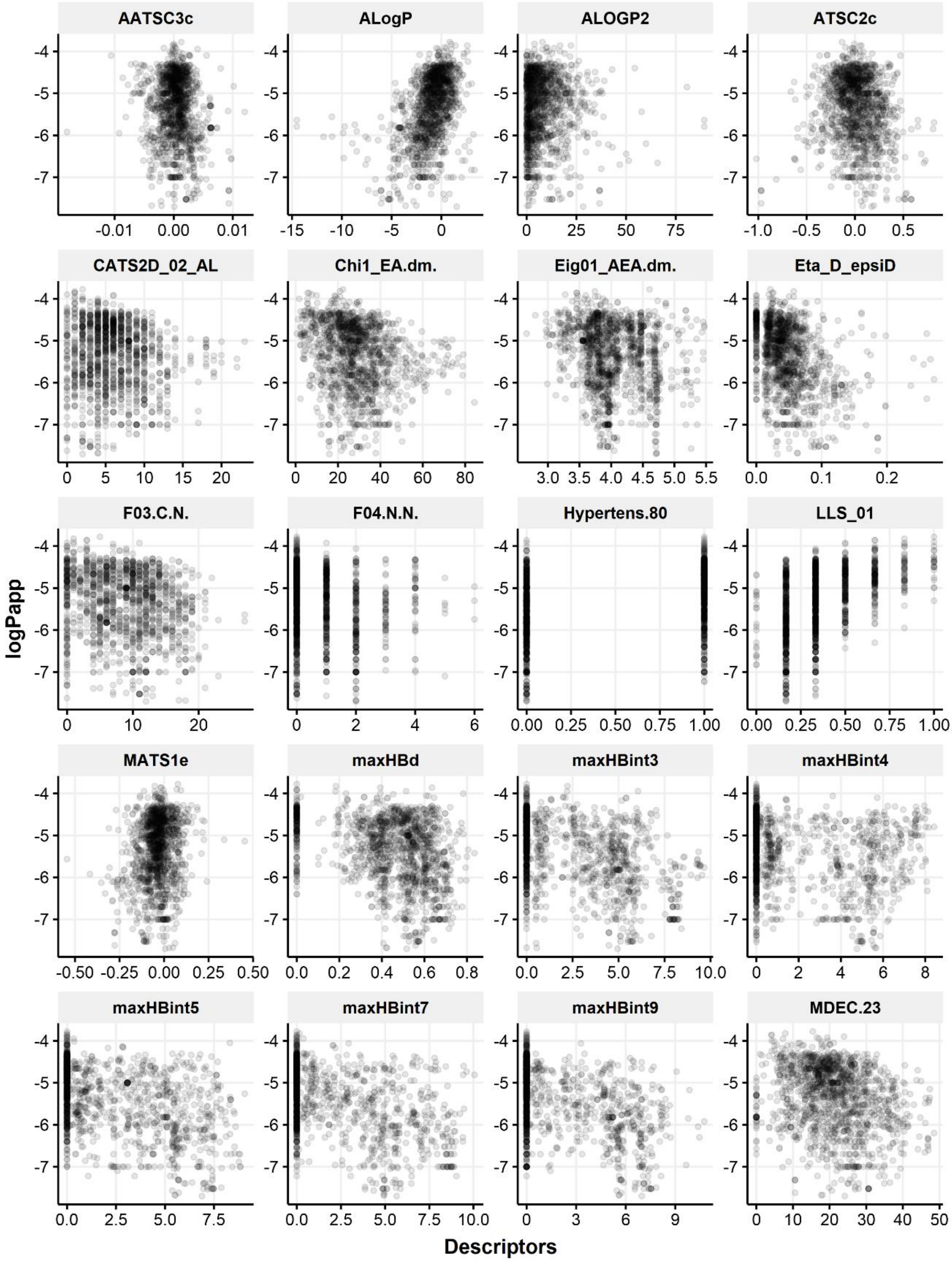

Figure S6. Correlation analysis between experimental logPapp values and selected molecular descriptors in the modeling dataset.

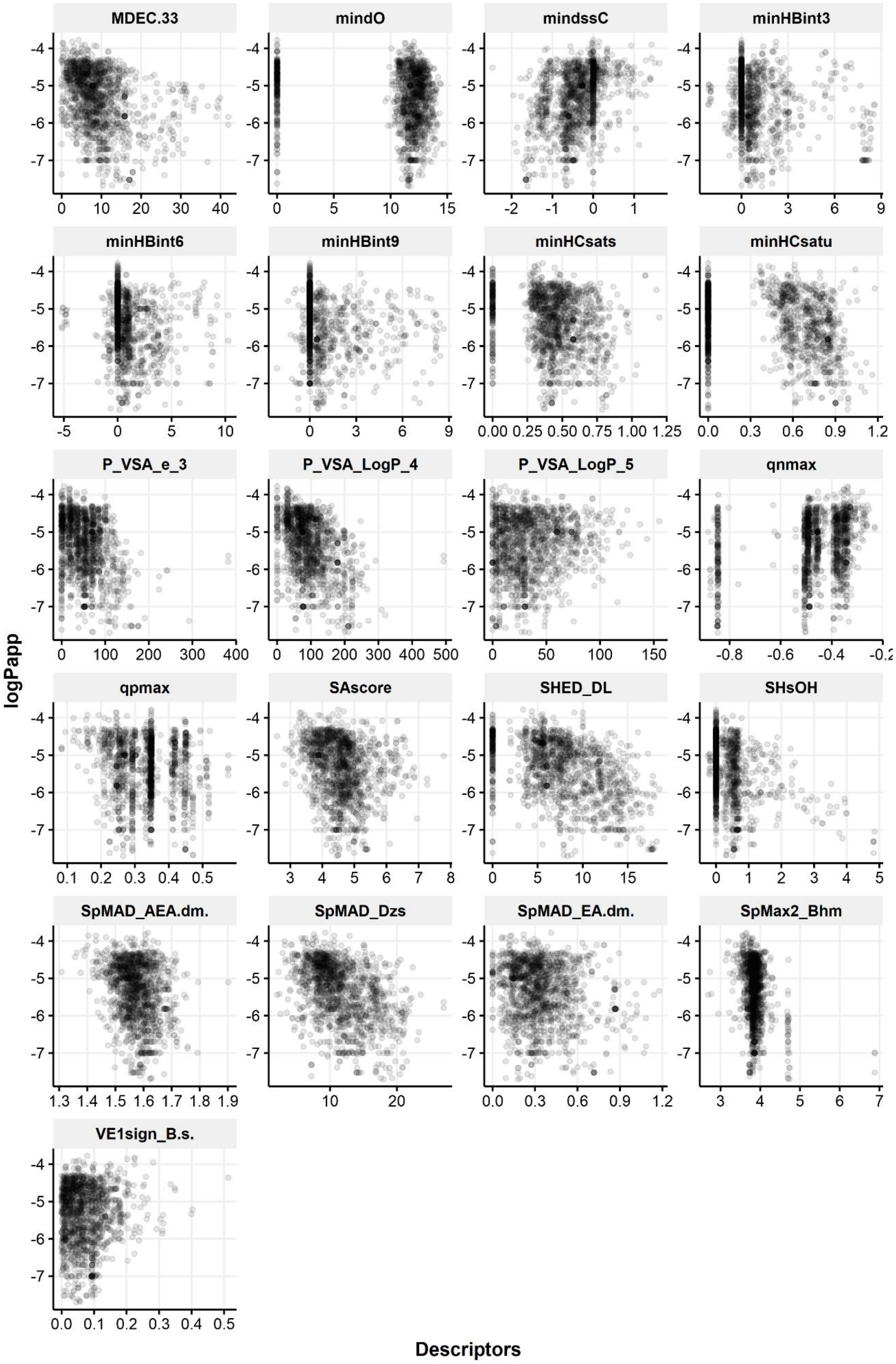

Figure S7. Correlation analysis between experimental logPapp values and selected molecular descriptors in the modeling dataset.
